# Neuronal antibodies in adult patients with new‐onset seizures: A prospective study

**DOI:** 10.1002/brb3.1442

**Published:** 2019-10-06

**Authors:** Johan Zelano, Markus Axelsson, Radu Constantinescu, Clas Malmeström, Eva Kumlien

**Affiliations:** ^1^ Department of Clinical Neuroscience Sahlgrenska Academy University of Gothenburg Gothenburg Sweden; ^2^ Department of Neurology Sahlgrenska University Hospital Gothenburg Sweden; ^3^ Laboratory for Clinical immunology Sahlgrenska University Hospital Gothenburg Sweden; ^4^ Department of Neurology Uppsala University Uppsala Sweden

**Keywords:** autoimmune, epilepsy, seizure

## Abstract

**Objectives:**

Immunotherapy in addition to antiepileptic drugs can improve seizure freedom rates in autoimmune epilepsy, highlighting the importance of early diagnosis. A diagnosis of autoimmune epilepsy can be supported by presence of serum antibodies to neuronal antigens. We asked how often neuronal antibodies are found in the serum of unselected adult patients with new‐onset seizures and whether such testing could improve detection of autoimmune epilepsy.

**Material and Methods:**

We included 44 patients over the age of 25 presenting after at least one unprovoked seizure to the Neurology Clinic at Sahlgrenska University Hospital, Gothenburg, Sweden. The median time between the first‐ever seizure in life and the serum sampling was 50 days (range 22–11,000). Antibody testing in serum was performed according to the manufacturer's instructions. The patients were followed for at least 1 year.

**Results:**

Epilepsy could be diagnosed already at the first visit in 21/44 patients (47.7%). Two patients (4.5%) were positive for neuronal antibodies: one against contactin‐associated protein 2 (CASPR‐2) and one against glutamate acid decarboxylase (GAD). Three patients (6.7%) displayed very weak immunoreactivity that was deemed clinically insignificant. One of the antibody‐positive patients had only a single seizure. The other had a focal cortical dysplasia and was seizure‐free on levetiracetam. None of the five patients with antibodies or immunoreactivity displayed any feature of autoimmune epilepsy.

**Conclusions:**

We conclude that indiscriminate testing in patients presenting to a first seizure clinic with new‐onset seizures or epilepsy is unlikely to improve detection of autoimmune epilepsy.

## Graphical abstract

We asked how often neuronal antibodies are found in the serum of unselected adult patients with new‐onset seizures and whether such testing could improve detection of autoimmune epilepsy. Two patients (4.5%) were positive for neuronal antibodies and three had weak immunoreactivity deemed clinically insignificant, none had clinical features of autoimmune epilepsy. We conclude that indiscriminate testing in patients presenting to a first‐seizure clinic with new‐onset seizures or epilepsy is unlikely to improve detection of autoimmune epilepsy.

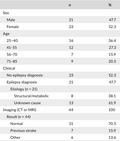

## 1 INTRODUCTION

Autoimmune epilepsy refers to epilepsy resulting from immune‐driven processes in the brain and is a recognized etiological category in the International League Against Epilepsy (ILAE) classifications of epilepsies (Scheffer et al., 2017). The clinical spectrum is probably wide, ranging from limbic encephalitis with prominent psychiatric symptoms to epilepsy without other symptoms than seizures. Nonparaneoplastic cases are more common than paraneoplastic ones, and autoimmune epilepsy has been suggested to account for up to 20% of epilepsy of unknown etiology (Bien & Holtkamp, 2017; Dubey, Alqallaf, et al., 2017; Irani, Bien, & Lang, 2011).

A diagnosis of autoimmune epilepsy can be supported by presence of neuronal antibodies in serum, which are most commonly directed against particular neuronal antigens (Irani et al., 2011). Antibody testing is usually prompted by clinical suspicion based on high seizure frequency, simultaneous onset of psychiatric symptoms, or particular seizure types like faciobrachial dystonic seizures. In cases of immune‐mediated epilepsy, immunotherapy in addition to antiepileptic drugs can improve seizure freedom rates, highlighting the importance of early diagnosis (Feyissa, Lopez Chiriboga, & Britton, 2017).

Studies on the prevalence of neuronal antibodies in patients with epilepsy of unknown etiology have yielded somewhat conflicting results. In two large epilepsy cohorts, neuronal antibodies were detected in serum in approximately one tenth of cases (Brenner et al., 2013; Suleiman et al., 2013). A higher rate was described in a recent prospective study, where investigators found antibody positivity in serum of more than one third (13/35) of adult patients with new‐onset epilepsy and an association between reduction in seizure frequency and immunotherapy (Dubey, Alqallaf, et al., 2017). In a more selected patient group with temporal lobe epilepsy, serum antibodies were found in 5.5% and a response to immunotherapy measured as a >50% reduction in seizures was seen in half of the antibody‐positive patients (Elisak et al., 2018).

Given the possibility of treatment response to immunotherapy, autoimmune epilepsy should ideally be found as early as possible. The potential of early identification by antibody testing was recently evaluated in a cohort of children with new‐onset seizures, in which very low rates of serum antibodies were detected (Garcia‐Tarodo et al., 2018). We are unaware of any study investigating the potential of early antibody testing to identify autoimmune epilepsy in adults. We therefore asked how often neuronal antibodies are found in the serum of unselected adult patients with new‐onset seizures and whether the presence of antibodies was associated with detection of autoimmune epilepsy.

## 2 MATERIALS AND METHODS

### 2.1 Study design and cohort

From June 2016, adult patients with new‐onset seizures, seen at the Department of Neurology at Sahlgrenska university hospital in Gothenburg, Sweden, were invited to participate in a prospective observational study evaluating biomarkers and quality of life in adult‐onset epilepsy. Recruitment was not systematic, and no screening log was kept. Sahlgrenska University Hospital is the largest neurology service in Gothenburg (population 650,000), providing secondary and tertiary epilepsy care. Most patients were seen within the setting of a “first seizure” clinic, where follow‐up is provided to patients referred from emergency departments. Inclusion criteria were a first unprovoked seizure after the age of 25, as assessed by the clinician at the visit (since the purpose was to test clinical utility). Exclusion criteria were progressive causes of the seizure‐like tumor or dementia and inability to give informed consent. Until December 2017, 44 patients had been included.

### 2.2 Antibody testing

Serum samples were collected at recruitment and stored in an approved storage facility (biobank, registration no 532) until analysis. Antibody analysis was performed with indirect immune fluorescence BIOCHIP encephalitis Mosaic (glutamate receptor type NMDA, glutamate receptor type AMPA ½, LGI1, CASPR2, GABA‐B receptors, and DPPX) and Euroline immunoblot (Tr, GAD65, Zic4, Titin, SOX1, Recoverin, Hu, Yo, Ri, Ma2/Ta, CV2, Amphiphysin), on automated Euroblot‐One instrument and results scanned EUROLineScan. Positive samples proceeded to indirect immunofluorescences on primate cerebellar/intestine slides BIOCHIP for confirmation according to the manufacturer's instructions (Euroimmune).

### 2.3 Clinical characteristics

At inclusion and at least 1 year after the collection of serum samples, patients were contacted by telephone or interviewed at a clinic visit and information was collected from the answers and the electronic medical record into a predefined clinical report form. Epilepsy was categorized according to a simplified scheme as either structural/metabolic or unknown cause. Imaging with CT is typically done before the patient is seen and MRI performed if an epilepsy cause is not identified on CT.

### 2.4 Statistical analyses

Data are expressed as median and range for continuous variables and frequencies for categorical variables. All analyses were performed using IBM SPSS version 23.

### 2.5 Ethics

All patients provided written informed consent. The regional ethical review board of Gothenburg approved the study, approval no 844‐15.

## 3 RESULTS

### 3.1 Cohort

The study cohort consisted of 44 patients seen in a first seizure clinic. Demographic and clinical characteristics are presented in Table 1. Approximately half of the patients did not meet epilepsy criteria, meaning that they had had only one seizure and a seizure recurrence risk estimated below 60% by the treating physician at the time of the serum sampling. The median time between the first‐ever seizure in life and the serum sampling was 50 days (range 22–11,000 days, the upper range being influenced by one patient having had a childhood seizure). Epilepsy was diagnosed already at the first visit in 21 patients.

**Table 1:** Study cohort

	*n*	%
Sex		
Male	21	47.7
Female	23	52.3
Age		
25–40	16	36.4
41–55	12	27.3
56–70	7	15.9
71–85	9	20.5
Clinical		
No epilepsy diagnosis	23	52.3
Epilepsy diagnosis	21	47.7
Etiology (*n* = 21)		
Structural/metabolic	8	38.1
Unknown cause	13	61.9
Imaging (CT or MRI)	44	100
Result (*n* = 44)		
Normal	31	70.5
Previous stroke	7	15.9
Other	6	13.6

Note

Demographics, clinical characteristics, and test results available at enrollment in the study cohort.

### 3.2 Antibody testing

Two patients (4.5%) were positive for neuronal antibodies: one against contactin‐associated protein 2 (CASPR‐2) and one against glutamate acid decarboxylase (GAD). Three patients (6.7%) displayed very weak immunoreactivity that was deemed clinically insignificant for Sox‐1 or Yo.

### 3.3 Seizure outcome

No patient with antibody reactivity was lost to follow‐up. Clinical course of all patients with neuronal antibodies or weak immunoreactivity is presented in Table 2. The patient with CASPR2 antibodies did not have any seizure relapse after the first seizure. Screening for malignancy revealed a localized asymptomatic sigmoid carcinoma. The patient with GAD antibodies had a cortical dysplasia on MRI, negative work‐up for malignancy, and remained seizure‐free on a low dose of levetiracetam. No patients with unspecific immunoreactivity had further tonic–clonic seizures; one patient had only a single seizure, one was diagnosed with juvenile myoclonic epilepsy (JME) based on emergence of myoclonia and EEG‐findings, and one had poststroke epilepsy. Both patients with epilepsy were seizure‐free on levetiracetam and had no clinical features suggestive of autoimmune epilepsy.

**Table 2:** Patients with neuronal antibodies or weak immunoreactivity

Patient no	Antibody	Malignancy	Epilepsy at enrollment	MRI	EEG	Further seizures	Medically refractory	Diagnosis at follow‐up
1	GAD	No	Yes	Focal dysplasia	Normal	No	No	Focal epilepsy
2	CASPR‐2	Sigmoid carcinoma	No	DWI: small silent ischemia	Normal	No	No	Single seizure
Weak reactivity								
3	(Sox1)	No	Yes	Calcification basal ggl	PSW	No	No	JME
4	(Sox1)	No	No	DWI: small silent ischemia	Normal	No	No	Single seizure
5	(Yo)	melanoma	Yes	Infarction	N/A	No	No	Focal epilepsy

Abbreviations: DWI, diffusion weighted imaging; JME, juvenile myoclonic epilepsy; PSW, poly spike wave.

## 4 DISCUSSION

In the present study, we investigated the presence of neuronal antibodies in unselected patients with onset of seizures after the age of 25. Only two out of 44 patients had such antibodies, and their presence was not associated with a severe epilepsy phenotype or prognosis. In fact, one patient had only one seizure and the other was seizure‐free on a low dose of an antiepileptic drug. Based on our findings, it would seem that antibody testing in unselected adult patients with new‐onset seizures is not justified. Our results also confirm that antibody‐positivity is not always linked to a severe epilepsy phenotype. In fact, in unselected patients with new‐onset seizures, presence of neuronal antibodies seems more likely to be a coincidental finding than a cause of the seizure.

Our findings are in agreement with recent reports from unselected children with new‐onset seizures, in which 1.9% had unspecific voltage‐gated potassium channel (VGKC)‐complex reactivity and 3.9% had reactivity to mouse neuropil (Garcia‐Tarodo et al., 2018). Our findings contrast somewhat to those in another adult cohort, in which 13 of 35 (37%) of patients with new‐onset epilepsy of unknown etiology were antibody‐positive (Dubey, Alqallaf, et al., 2017). Importantly, we included patients with new‐onset single seizures in our cohort, which may explain part of the difference. In our study, 8 out of the 44 patients had epilepsy of structural or metabolic origin (6 prior stroke, 2 other structural abnormality) at the time of enrollment. Even if these are excluded the proportion of antibody‐positive patients in, our cohort is still only 5.5% (two patients). Importantly, no signs of autoimmune epilepsy emerged during follow‐up in these two patients. One of the patients had serum GAD antibodies, which has been detected in 5.5% of JME patients and 4% of healthy controls in a study where the authors, just like in our study, found no association between antibody‐positivity and poor epilepsy outcome (Aykutlu, Baykan, Gurses, Gokyigit, & Saruhan‐Direskeneli, 2005).

The role of antibody testing in the field of epilepsy is not yet clear. The aim of our study was not to investigate the prevalence of specific antibodies or of autoimmune epilepsy—for that purpose single centre recruitment is far from sufficient and a much closer clinical characterization of all patients would be needed. Our aim was instead to evaluate whether extensive antibody testing applied in a first seizure clinic could improve detection of autoimmune epilepsy. This does not seem to be the case. After the design of this study, clinical algorithms like the APE score for improving pretest probability have been developed and our results argue in favor of their utilization (Dubey, Singh, et al., 2017; Graus et al., 2016). We conclude that indiscriminate antibody testing in patients presenting to a first seizure clinic with new‐onset seizures or epilepsy seems unlikely to improve detection of autoimmune epilepsy. More sophisticated pretesting selection is probably required.

## CONFLICT OF INTEREST

JZ has received consultancy fee from the Swedish Medical Products Agency and writers honoraria from the journal Neurology in Sweden, and is as an employee of Sahlgrenska university hospital investigator/subinvestigator in clinical trials sponsored by GW Pharma, SK life science, and Bial (no personal compensation). MA has received compensation for lectures and/or advisory boards from Biogen, Genzyme, and Novartis. CA has received honoraria for lectures and advisory boards from Biogen and Novartis.

## Data Availability

Some anonymized data that support the findings of this study can be shared by the corresponding author upon request. All data are not publicly available due to confidentiality/privacy restrictions.
